# Systematic Comparison of Experimental Crystallographic Geometries and Gas-Phase Computed Conformers for Torsion Preferences

**DOI:** 10.1021/acs.jcim.3c01278

**Published:** 2023-11-24

**Authors:** Dakota L. Folmsbee, David R. Koes, Geoffrey R. Hutchison

**Affiliations:** † Department of Chemistry, University of Pittsburgh, 219 Parkman Avenue, Pittsburgh, Pennsylvania 15260, United States; ‡ Department of Anesthesiology & Perioperative Medicine, School of Medicine, University of Pittsburgh, Pittsburgh, Pennsylvania 15261, United States; § Department of Computational & Systems Biology, School of Medicine, University of Pittsburgh, Pittsburgh, Pennsylvania 15260, United States; ∥ Department of Chemical & Petroleum Engineering, University of Pittsburgh, 3700 O’Hara Street, Pittsburgh, Pennsylvania 15261, United States

## Abstract

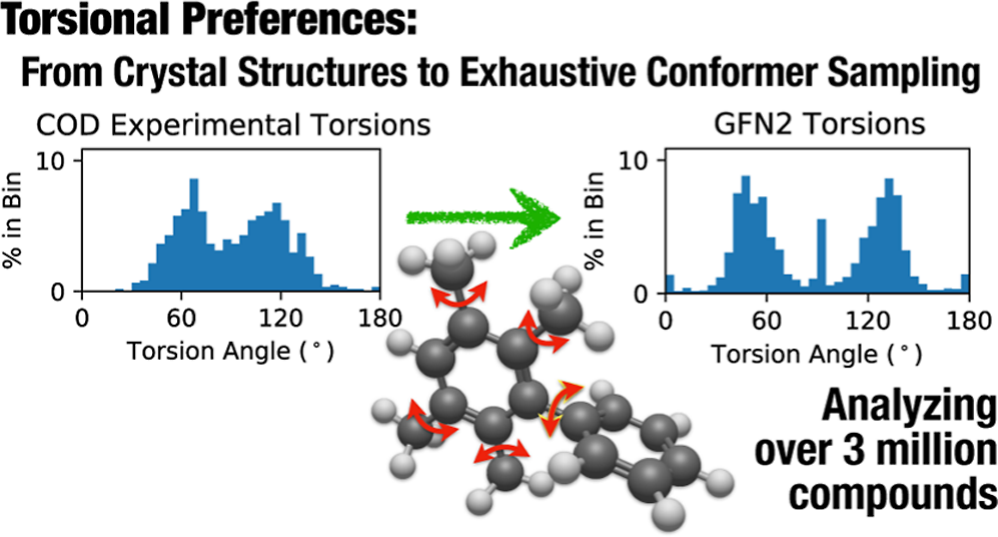

We performed exhaustive torsion sampling on more than 3 million compounds using the GFN2-xTB method and performed a comparison of experimental crystallographic and gas-phase conformers. Many conformer sampling methods derive torsional angle distributions from experimental crystallographic data, limiting the torsion preferences to molecules that must be stable, synthetically accessible, and able to be crystallized. In this work, we evaluate the differences in torsional preferences of experimental crystallographic geometries and gas-phase computed conformers from a broad selection of compounds to determine whether torsional angle distributions obtained from semiempirical methods are suitable priors for conformer sampling. We find that differences in torsion preferences can be mostly attributed to a lack of available experimental crystallographic data with small deviations derived from gas-phase geometry differences. GFN2 demonstrates the ability to provide accurate and reliable torsional preferences that can provide a basis for new methods free from the limitations of experimental data collection. We provide Gaussian-based fits and sampling distributions suitable for torsion sampling and propose an alternative to the widely used “experimental torsion and knowledge distance geometry” (ETKDG) method using quantum torsion-derived distance geometry (QTDG) methods.

## Introduction

Most molecules exhibit some level of conformational flexibility, the existence of multiple low-energy geometries that differ mostly by changes in the torsional angles of both acyclic and ring bonds. Many methods have been developed to sample conformations, with benchmarks frequently focusing on finding one geometry close to an experimental crystal structure.^1−4^ Consequently, most conformer sampling methods derive torsional angle distributions from experimental crystallographic data^1,5−8^ not only to provide geometries close to such benchmarks but also as large diverse repositories of “ground truth” geometric properties such as bond lengths, angles, and dihedrals.^9−11^

One challenge is that experimental crystallographic data are limited by the size of the data source^12^ and reflect some inherent biases. In order to be collected, the molecules must be stable, synthetically accessible, and actually made and crystallized. While new cryo-electron microscopy (cryo-EM) techniques are improving dramatically and have less stringent requirements on crystals, generally, growing high-quality crystals for small-molecule crystallography is a time-consuming process. Moreover, it is known that compounds with experimental crystal structures are generally smaller and exhibit fewer conformers than other compounds.^13^ Similarly, compounds containing elements outside the common organic subset (e.g., B, As, and Se) or less common chemical motifs may be poorly represented in experimental crystallographic databases. Also, even for compounds found in an existing database, much chemistry is performed in solution and gas phases, where solid-state preferences may not directly apply.^14−18^ Finally, several works have noted challenges with deriving data from some crystallographic databases.^12,19^

Consequently, finding unbiased alternative sources of accurate and reliable torsional angle preferences could significantly expand the use of conformational sampling to a new chemical space. Typically, sampling has been performed using small-molecule force fields (e.g., UFF^20^), which have shown limited fidelity compared to density functional and other first-principles quantum chemical methods.^21,22^ The development of efficient dispersion-corrected semiempirical methods such as GFN2-xTB,^23^ as well as new machine learning methods such as ANI^24−27^ and OrbNet,^28,29^ offers improved accuracy of torsional angles and nonbonded interactions with moderate computational cost. Moreover, several large-scale computational efforts including PubChemQC^30^ and the QCArchive^31,32^ have provided large amounts of optimized gas-phase theoretical geometries using high-quality density functional methods.

In this work, we outline an extensive effort to analyze the conformers and torsional angle preferences of more than 3 million organic small molecules, using exhaustive sampling using the GFN2-xTB method across both the experimental Crystallographic Open Database^33^ (COD) and multiple sets of small molecules, including PubChemQC.^30^ We compare the potential bias between the crystallographic and gas-phase geometries and individual torsion patterns, including analysis with ωB97X-D3^34^ with the def2-SVP basis set.^35,36^

## Methods

Molecules for this work were compiled from several sources, including 88,106 organic compounds from the Crystallographic Open Database,^9,10^ 3,009,591 molecules from PubChemQC,^30^ 88,550 molecules from the Pitt Quantum Repository, and previous work on conformational flexibility, which included 70,850 molecules from a subset of ZINC^37^ and 4378 molecules from the Platinum ligand database.^3^ For all sources, the largest substructure was retained (i.e., the solvent or salts were removed from the crystallographic unit cells). For compounds without initial 3D coordinates, Open Babel 3.1^38−40^ was used to generate initial coordinates, since CREST requires an initial geometry. As noted above, the total set of compounds included more than 3 million unique molecules. Properties across the data sets are illustrated in Figures S1–S4 and are roughly comparable to the Platinum Diverse set and the DrugBank-approved set,^41^ with the exception that the PubChemQC set compounds are smaller.

For each molecule, conformers were generated using the CREST program to exhaustively sample the potential energy surface, using default parameters and the GFN2-xTB method to compute energies (hereafter known as simply GFN2) and optimize geometries.^23,42,43^ In some cases, CREST produced fragments or chemical rearrangements (e.g., producing different compounds than the input, based on the InChI identifier)—these systems were excluded from analysis. While, in principle, CREST can generate many conformers per compound, we find that for the vast majority of compounds, only a few conformers are generated within 6 kcal/mol as calculated by GFN2 (Figures S5 and S6), consistent with our previous work.^44^

In this work, the lowest-energy conformer by GFN2 energy was analyzed. Torsional angle SMILES arbitrary target specification (SMARTS) patterns from the experimental torsion knowledge-based distance geometry (ETKDG) approach^6−8^ were used to generate the histograms for the gas-phase data. ETKDG derives torsional preference distributions for the RDKit distance geometry coordinate generation method from the analysis of experimental crystallographic data for a set of hierarchical dihedral patterns.^6−8^

These patterns are constructed from molecules with central bonds of C–C (168 acyclic patterns), C–O (56 acyclic patterns), C–S (16 acyclic patterns), N–C (131 acyclic patterns), N–S (4 acyclic patterns), and S–S (1 pattern) and create the 387 acyclic and 105 ring dihedral SMARTS patterns used to generate histograms of matching torsions with a stepsize of 5° using RDKit^45^ Python scripts (see the Supporting Information). Figures depicting the SMARTS patterns were generated using SMARTS.plus.^46,47^

For selected compounds, to compare the GFN2 geometry with density functional theory (DFT), optimization was performed with ORCA 4.2.0^48^ using the ωB97X-D functional^34^ and the def2-SVP basis set,^35,36^ which has proven to produce fairly accurate conformational energetics^21,49,50^ although some errors still exist when compared with more accurate methods.^51−54^

## Results and Discussion

Traditionally, conformer sampling is refined via classical force fields, which yield a poor correlation with energies from more accurate quantum methods.^21,22^ On the other hand, geometry optimization with most density functional methods requires hours per conformer, making large-scale sampling prohibitive. Recently, larger data sets have emerged, including this work, as well as the ANI-1x,^55^ GEOM,^56^ QCArchive,^31,32^ QMugs,^57^ and SPICE sets.^58^ The goal of this work is to show that while gas-phase quantum chemical geometries require substantial time to generate, such data sets can be used to augment or supplant traditional crystal structure sources to establish torsional preferences.

Below, we will consider the overall molecular geometries between optimized gas-phase conformer ensembles and experimental crystal structures, individual torsional distributions, compare the GFN2-optimized and DFT-optimized geometries, and finally fit the torsional distributions via Fourier analysis or sets of Gaussian peaks.

### Comparing Overall Geometries

Often, coordinate generation and conformer tools are evaluated, in part, by comparing the ensemble of generated geometries to experimental crystallographic geometries. For example, the ETKDG method has proven to generate structures with small root-mean-square displacement (RMSD) when compared to small-molecule crystal structures and bound-ligand geometries.^1,3,6,7^

Consequently, our first comparison is between the CREST-generated GFN2 conformer ensembles and ETKDG 250 conformer ensembles across the Crystallographic Open Database (COD) and Platinum Diverse data sets. As noted above, while CREST attempts to generate exhaustive ensembles under 6 kcal/mol from the global minima, in general, only a few conformers are generated (Figures S5 and S6), and 250 conformers is sufficient to encompass 93% of the COD set and 85% of the Platinum Diverse set (Figure S5). While larger ensembles tend to yield smaller RMSD, a comparison is still useful despite some differences in ensemble size.^59^

**Figure 1:**
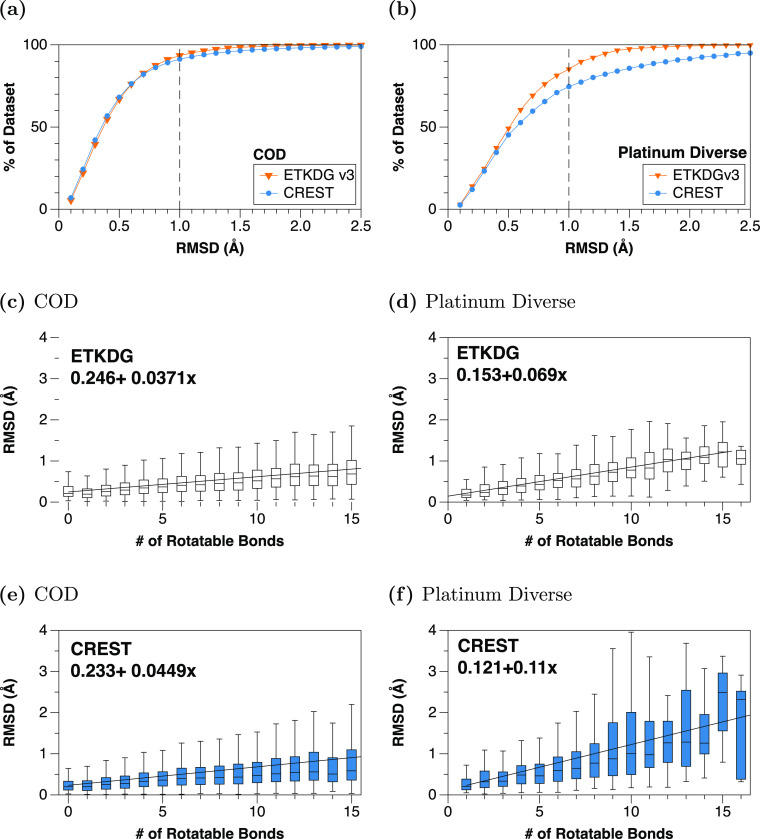
Comparison of the smallest non-hydrogen RMSD between experimental crystallographic geometry and CREST or ETKDG conformers for the (left) Crystallographic Open Database (COD) and (right) Platinum Diverse data set. (c–f) Captions indicate best-fit linear regression in Å. Note that for both data sets, CREST produces smaller RMSD for molecules with few rotatable bonds but a larger slope indicating generally worse RMSD for larger compounds with more rotatable bonds.

Calculating the smallest non-hydrogen RMSD of each generated ensemble and the experimental crystal structure geometry (Figure 1), we find that the CREST ensembles perform better than ETKDG on molecules with few rotatable bonds (e.g., from zero to ∼3–4 rotors) on the COD set, have comparable RMSD across a broader range of COD molecules, and perform slightly worse on the Platinum set (e.g., Figure 1a,b and Table S1). Note that the closest geometry to the experimental geometry is often not the lowest-energy CREST/GFN2 conformer.

As illustrated in Figure 1 and summarized in Tables S1 and S2, for the COD small-molecule crystal structures, the CREST/GFN2 method has a higher fraction of molecules within an RMSD of 0.2–0.5 Å, compared to ETKDG, and a smaller median RMSD. Such performance arises mostly from molecules up to 3–4 rotatable bonds. The improved treatment of nonbonded interactions and electrostatics in the quantum GFN2 method likely gives rise to these geometries more closely matching experimental crystallographic geometries.^21^

Note, however, that for the Platinum set, derived from bound PDB ligands, the performance of the CREST ensembles is generally worse than that of ETKDG, with a worse median and mean RMSD and a considerably higher RMSD increase with the number of rotatable bonds, as illustrated in Figure 1. We speculate that this difference derives from comparing gas-phase CREST/GFN2 conformers with bound-ligand geometries, which may be more stabilized in extended conformations due to intermolecular interactions with a binding site and solvent.^60^

**Figure 2:**
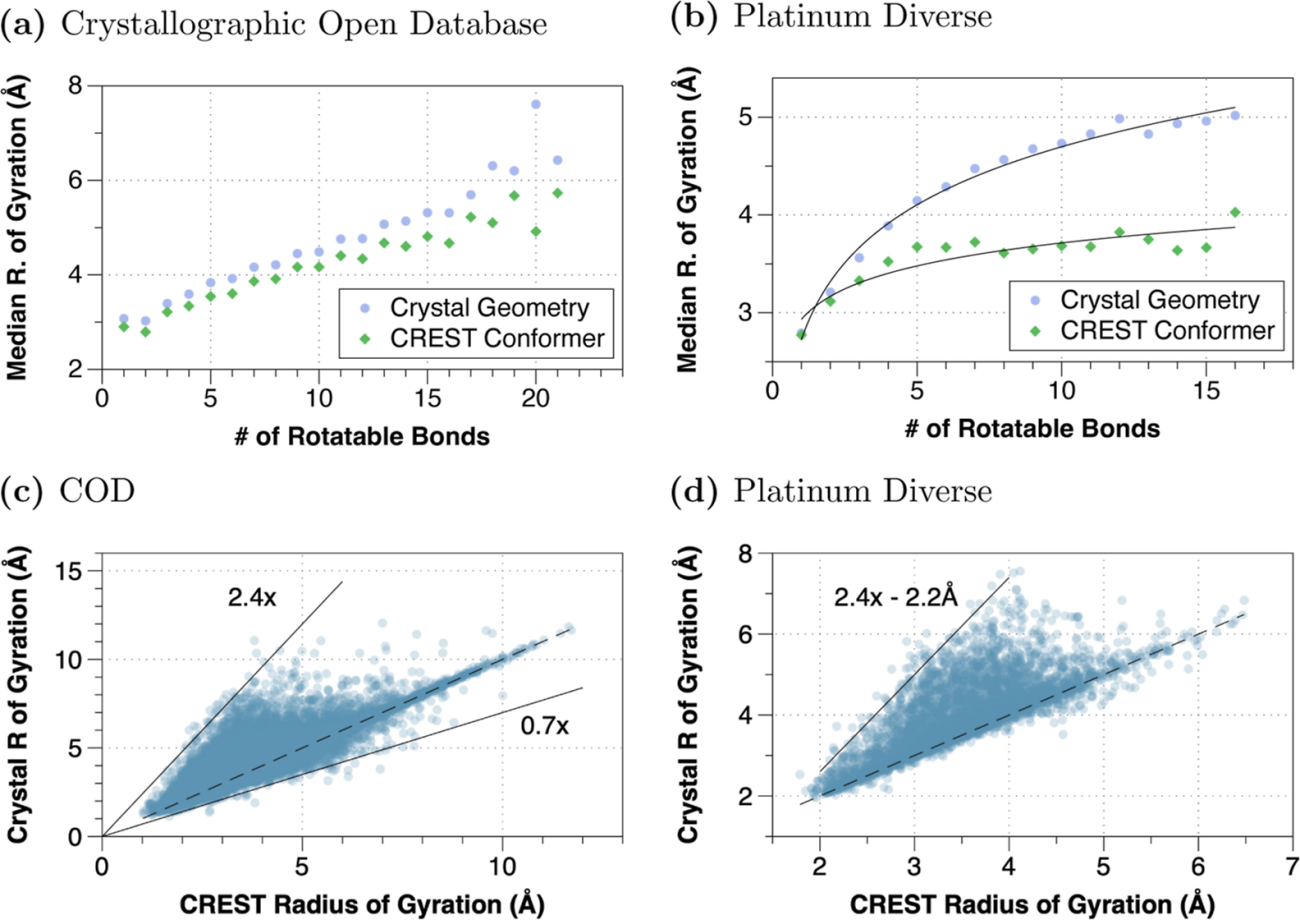
Calculated radius of gyration for the lowest-energy CREST conformer and experimental geometries as a function of the number of rotatable bonds for the (a) Crystallographic Open Database (COD) and (b) Platinum data sets and scatterplot of compounds from (c) COD and (d) Platinum sets, comparing the radius of gyration from the CREST/GFN2 lowest-energy conformation with that of the experimental crystal structure geometry. Dashed line indicates a 1:1 correspondence, with approximate bounds indicated by solid lines.

While there is a wide distribution of the calculated radius of gyration between the lowest-energy CREST/GFN2 generated conformations and the experimental crystal structures from COD and Platinum sets (Figure 2), as compiled in Figure 2, the median radii of gyration for experimental COD and CREST-generated conformers are relatively close (e.g., the ratio of the two is usually close to 1.0, Figure S7). On the other hand, the Platinum crystallographic geometries show a notably larger radius of gyration than the CREST conformer (e.g., Figure S7), with the deviation growing as a function of the number of rotatable bonds.

Overall, the results indicate that CREST-derived conformer ensembles perform comparably to ETKDG ensembles on the small-molecule COD set, with the caveat of a potential bias toward compact conformations relative to the Platinum bound PDB ligands, likely due to a neglect of intermolecular interactions in the gas-phase geometries.

While CREST gas-phase ensembles perform acceptably compared to ETKDG on the small-molecule COD set, the time required for the calculations is large, as illustrated in Figure S8. The median runtime across the COD set is 1 h and increases as *n*^2^(62) with *n* as the number of atoms as both the semiempirical GFN2 geometry optimizations increase in time and larger molecules generally yield more conformers (e.g., Figure S6). Consequently, the approach discussed below is to use a database of CREST ensembles to derive torsional preferences suitable for developing faster conformational search tools.

### Individual Torsion Preferences

As mentioned above, traditional efforts to derive torsional preferences use experimental crystallographic databases.^1,3,5−8^ Given the reasonable agreement between CREST/GFN2 conformers and crystallographic geometries, particularly on the small-molecule COD set, the individual torsion preferences should also be comparable. Thus, we compared the experimental torsions from the COD to the lowest-energy gas-phase CREST/GFN2 generated conformers from the COD, as well as to CREST/GFN2 generated conformers of all combined sets (e.g., PubChemQC, COD, etc.) across over 3 million compounds.

Comparing torsions between experimental geometries from the COD and lowest-energy gas-phase conformers from CREST/GFN2 is intended to show the suitability of gas-phase torsion preferences to replace or supplement standard crystallographic analysis. Poor correlation between individual torsion distributions could occur for a few main reasons—that the gas phase and experimental crystal structures show distinct angles due to intermolecular interactions and packing effects, that the distributions have few points and are thus inherently noisy, or that the GFN2 semiempirical method is not sufficiently accurate to reproduce torsion angles.^51,61^

However, the COD is significantly smaller than the Cambridge Crystallographic Database,^11,62^ which limits the number of torsion data points for some structural motifs. Expanding our analysis to include millions of molecules can improve the understanding of uncommon motifs as well as potential torsional preference differences in the solid state compared to the gas-phase geometries. This work will examine the comparison of crystal structure and calculated gas-phase torsional preferences for both ring- and acyclic-containing torsions.

**Figure 3:**
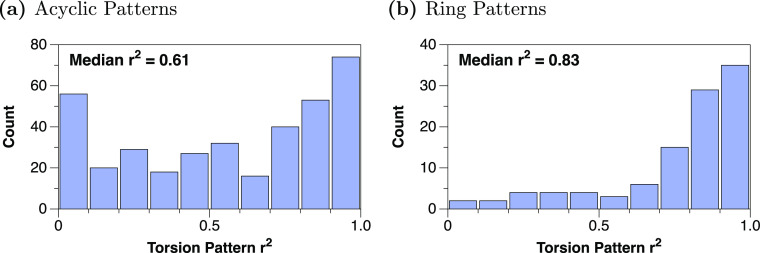
Correlation between experimental and gas-phase torsions across the COD data set for (a) acyclic patterns and (b) ring patterns.

To determine the degree of correlation between the COD experimental torsion preferences and the computed gas-phase preferences, the *r*^2^ of the kernel density estimation (KDE) for both acyclic and ring torsion patterns was compiled, as shown in Figure 3a. The KDE is used to smooth the histogram (i.e., representing uncertainty in the individual torsions) and to avoid issues when correlating areas with few or no torsions in the COD with the regions where some torsions were present in the combined set. The *r*^2^ then correlates the KDE of the experimental COD torsion preferences and the KDE of the computed CREST/GFN2 gas-phase preferences for each torsion pattern. A total of 127 patterns yield *r*^2^ > 0.8 and the median acyclic *r*^2^ is 0.61, indicating that while there are some differences, the torsional preferences yield a reasonable correlation.

The correlation is likely better indicated by the median value, as shown by analyzing the 76 patterns with an *r*^2^ less than 0.2. The patterns in this regime had a median of only 175 instances across the COD experimental set, which appears to be too few to form accurate distributions of the dihedral angle preferences.

In short, we believe that in most cases, the correlation between experimental crystallographic geometries and gas-phase conformer sampling with semiempirical quantum methods such as GFN2 is high enough to derive accurate torsional preferences. This is consistent with previous comparisons of energetic rankings of conformers between GFN2 and higher-level quantum chemical methods including density functional and coupled cluster calculations,^21^ as well as efforts to estimate torsional strain in crystal structures using DFT methods.^61,63,64^

Ring torsional patterns, constrained by the nature of a ring, show even greater correlation, with the median *r*^2^ at 0.83 (Figure 3b) and few patterns (16 out of 105) showing correlations below 0.5, again correlated with few matches.

**Figure 4:**
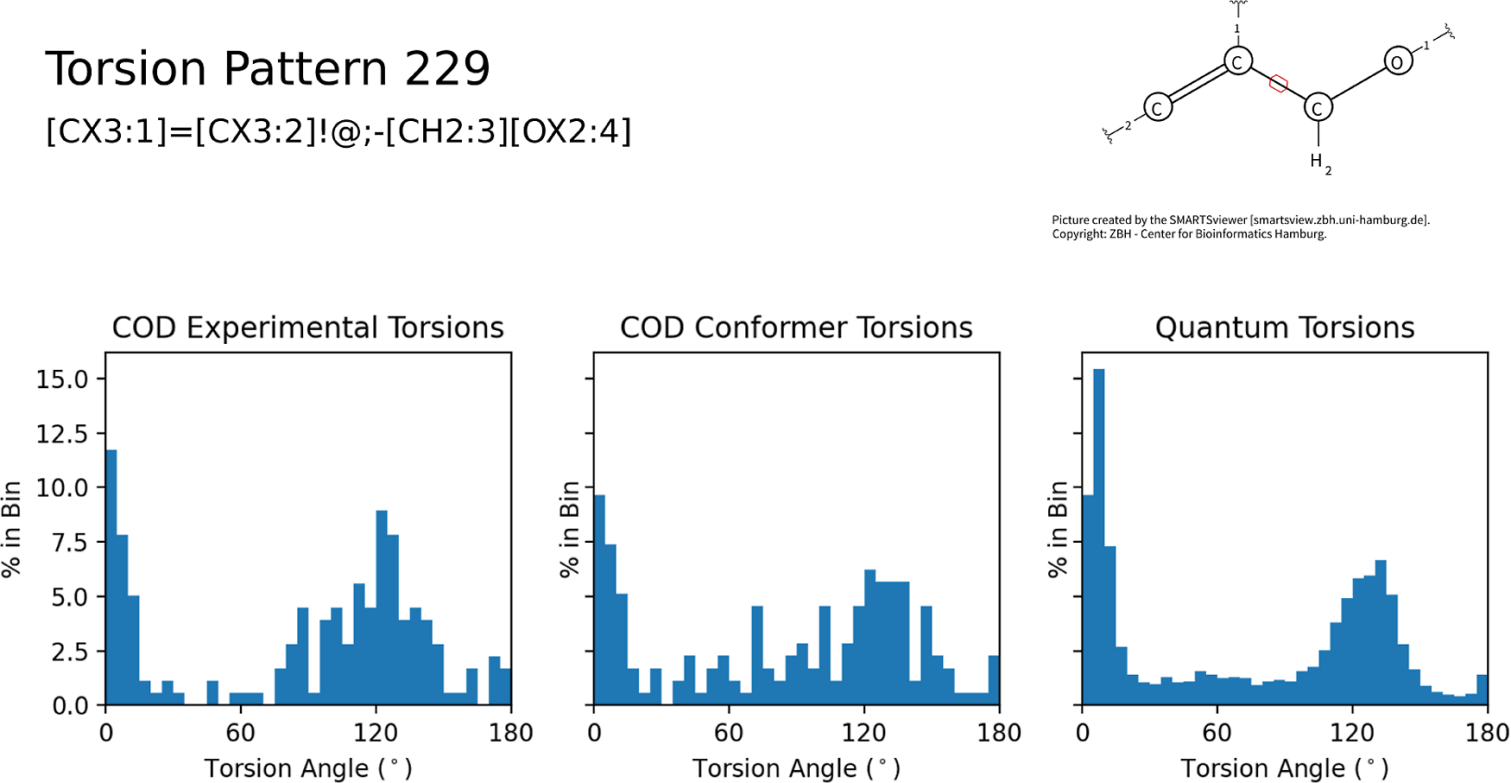
Histograms for pattern 229, including COD experimental torsional angles, gas-phase lowest-energy conformers from the same COD molecules, and gas-phase lowest-energy conformers across the entire data set, indicating the strong correlations and that the increased quantity of data greatly refines the histograms.

Individual acyclic torsional preferences can be further analyzed to determine the qualitative correlation between the crystal structures and gas-phase conformers. Torsion pattern 229 shown in Figure 4 demonstrates a high degree of correlation (*r*^2^ = 0.76) between the crystal structures and gas-phase CREST/GFN2 lowest-energy conformers while demonstrating the advantage of using additional data from the entire set of compounds. Pattern 229 has only 179 torsions from the COD, while the expanded data set boasts over 25,000 instances. This increase in data clarifies torsional preferences in the range of 90–150° as the conformers demonstrate a clearer peak in this region and less noise overall.

**Figure 5:**
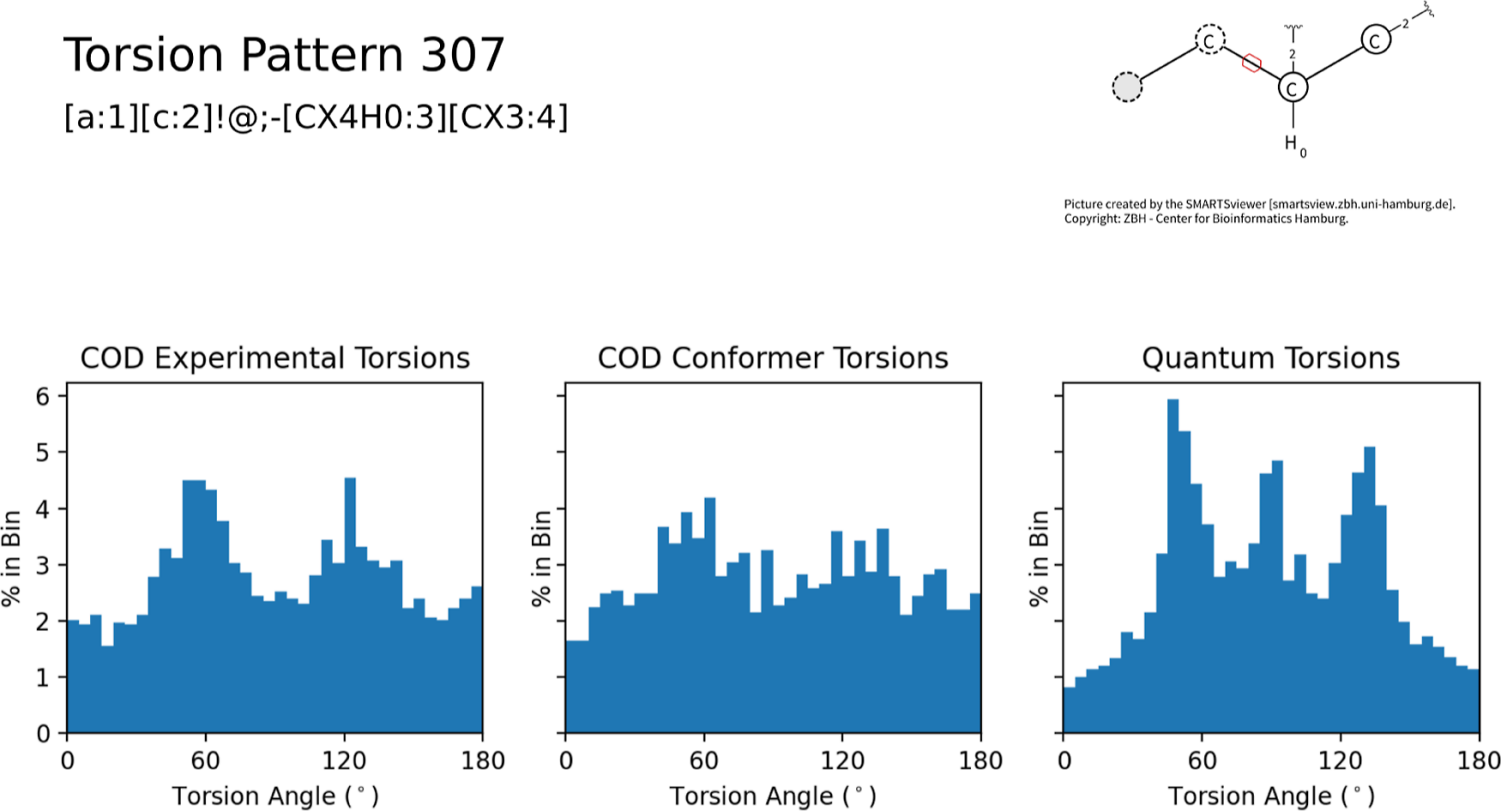
Histograms for pattern 307, including COD experimental torsional angles, gas-phase lowest-energy conformers from the same COD molecules, and gas-phase lowest-energy conformers across the entire data set, again indicating how the increased quantity of data greatly refines the torsional preferences (e.g., peaks near 50, 90, and 132°).

Figure 5 demonstrates how the lack of data in the COD can impact qualitative assessments of the torsional preferences. The correlation between the experimental COD and computed gas-phase torsions (left and middle panels) is 0.58. The experimental torsions show a mild preference around 60 and 120°, with a lot of noise between, while the calculated torsions have more defined peaks at 50, 90, and 132°. Much like the case above, the 10× increase in the number of torsions in the combined set was able to provide enough data to discern preferential angles.

In addition to the acyclic preferences, we analyzed the preferences of ring torsional patterns. Ideally, torsional patterns in rings should correlate well between experimental and calculated gas-phase geometries due to the steric constraints on the geometry. The correlation was analyzed in the same manner as above by taking the *r*^2^ of the kernel density estimation (KDE) for each ring torsion pattern and compiling them into Figure 3b. Compared with the median *r*^2^ of 0.61 for the acyclic patterns, the ring patterns boasted a median *r*^2^ of 0.83, indicating a significant correlation between the two sets.

**Figure 6:**
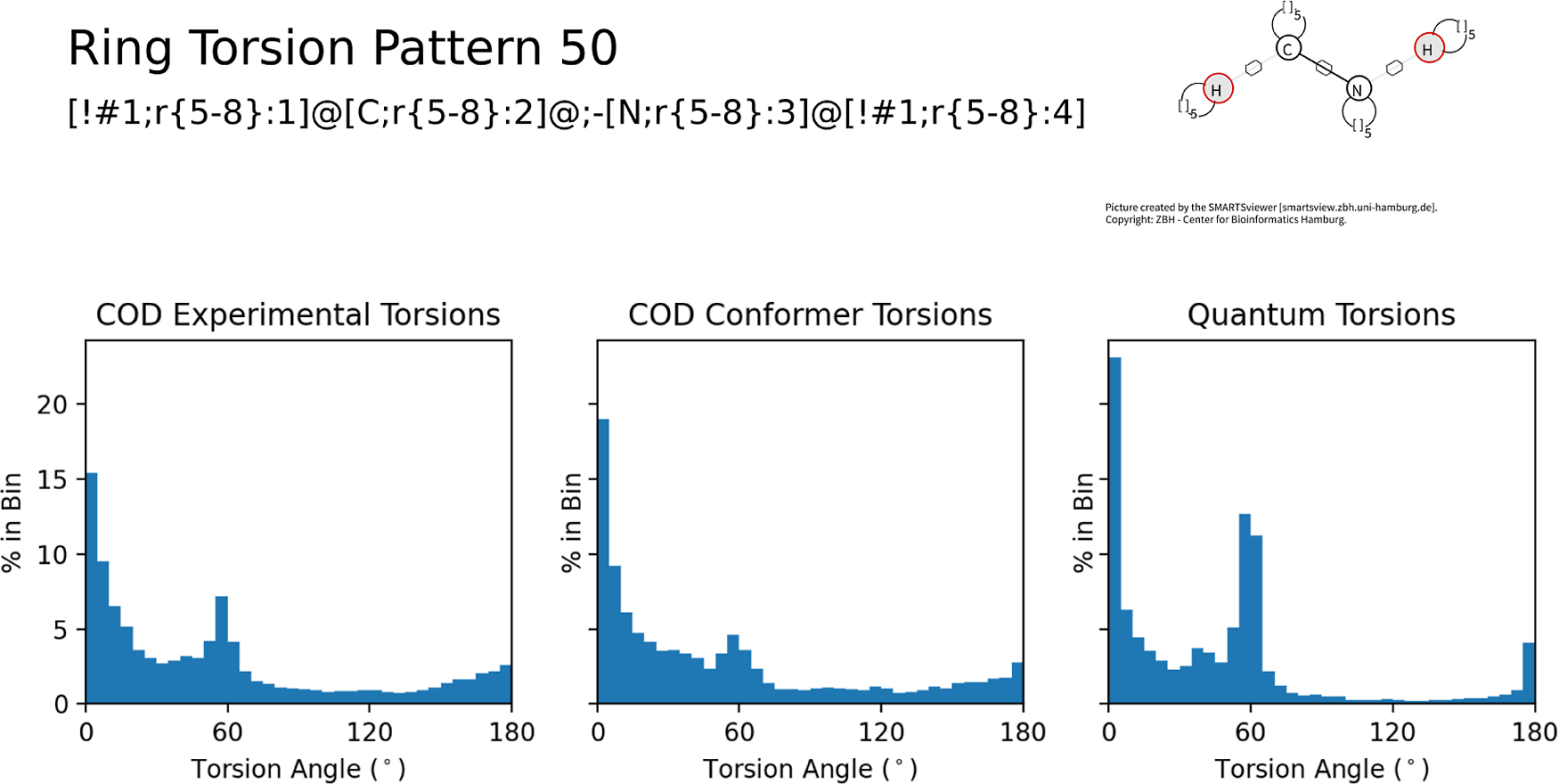
Histograms for COD experimental torsional angles, gas-phase lowest-energy conformers from the same COD molecules, and conformers across the entire data set, again indicating that the increased quantity of data greatly refines the torsional preferences (e.g., a strong peak at 60°).

Similar to the case for acyclic patterns, the increase in available data bolsters the angular preferences of the ring torsion patterns. Figure 6 exemplifies this by demonstrating how the increase in the number of torsions for ring torsion pattern 50 affects the torsional preference. The correlation between the experimental and gas-phase COD data is 0.79. Although the experimental data suggests 0, 60 and 180° are prominent angles; the additional data from the combined sets firmly demonstrates that these are prominent angles for the torsion pattern, with a small subpopulation at ∼41°. The addition of more data exhibits a more complete picture of torsional preferences that better represent the chemical space.

These expected similarities for the ring patterns are due to the intermolecular constraints that the ring structure imposes on the geometry. There is less flexibility in ring systems. The correlation of individual ring torsions demonstrates the accuracy of the quantum torsional preferences and indicates the ability to use this information as an additional method for determining the torsional preferences of structural motifs not yet examined through experimental means.

Importantly, sampling ring torsion angles in isolation is inherently challenging since such angles are strongly correlated with other torsion angles in the same ring.^65^ In the ETKDG method, such a correlation is resolved through the distance geometry methods, refining distances between all atoms in the ring (and molecule) together. As such, the ring torsion data in this work is intended either for refining such distance geometry methods or as an initial effort to refine ring puckering distributions using Cremer–Pople schemes.^65^

The outlined work demonstrates the desire for additional methods based on quantum calculations where crystal structure constraints may not be suitable or data may not be prevalent due to experimental limitations. Using the example of ETKDG,^7^ an alternative, quantum torsion distance geometry (QTDG) could be useful for gas-phase applications.^66^ Moreover, a QTDG method would no longer be constrained to structural preferences derived from what can be synthesized and crystallized. This allows for an increase in the capability of the method because a larger amount of data would be available for a more diverse representation of chemical space.

### Comparisons between GFN2- and DFT-Optimized Geometries

**Figure 7:**
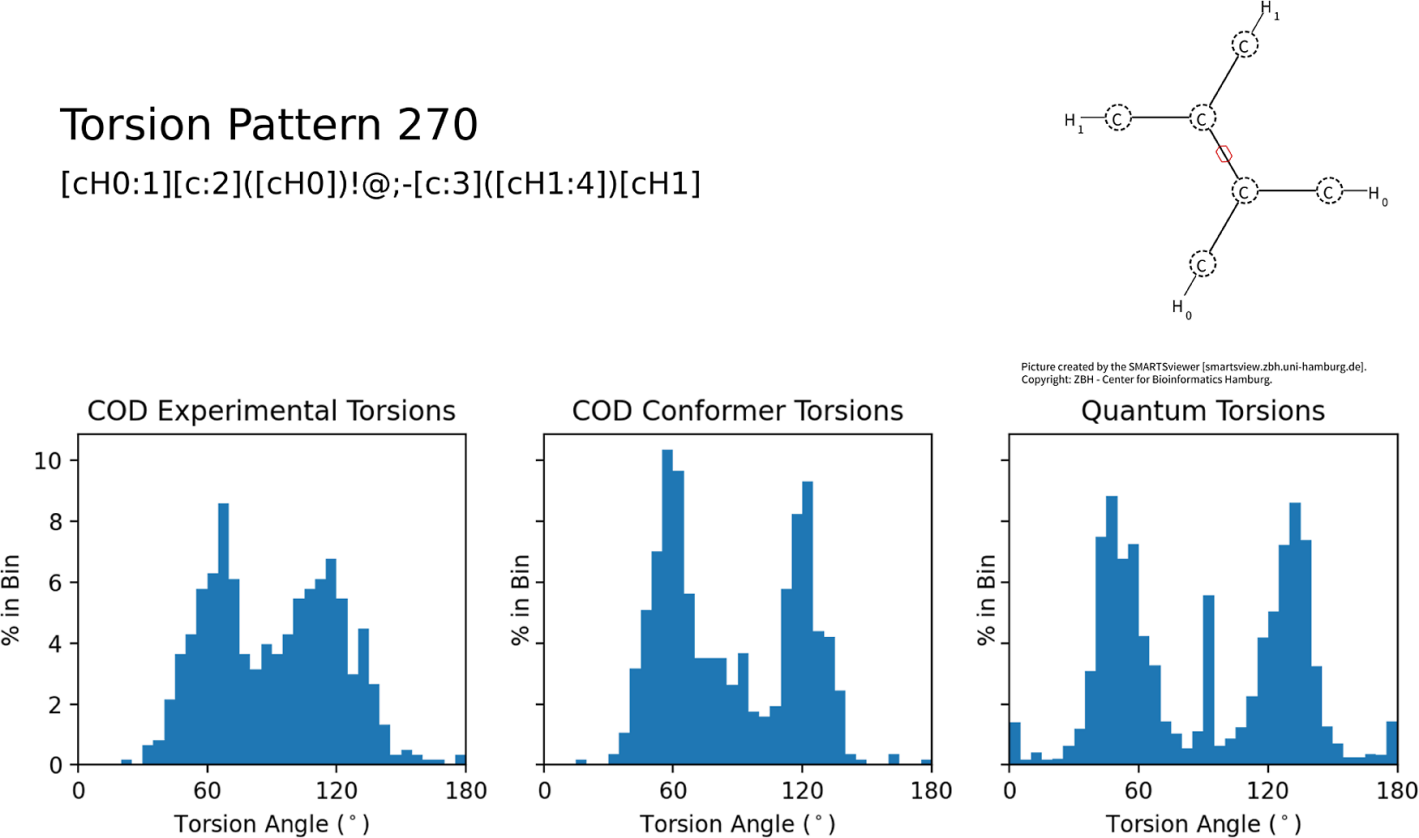
Differences in torsional preferences for experimental and gas-phase geometries.

As noted above, some differences between the COD crystallographic torsional preferences and those calculated from gas-phase CREST/GFN2 lowest-energy conformers derive from patterns with a few candidates and thus somewhat noisy histograms. In other cases, the histograms suggest noticeable differences involving new peaks, particularly those from the combined set. For example, in Figure 7, the experimental torsions show a mix of torsions in the range of 70–110°, while the calculated torsions from the combined set also show a dominant peak at 90°.

This new peak could derive from a new subpopulation, for example, compounds not present in the COD or not synthetically accessible or amenable to crystallization. The new peaks could also derive from problems with the GFN2 method, predicting incorrect torsion angles.

**Figure 8:**
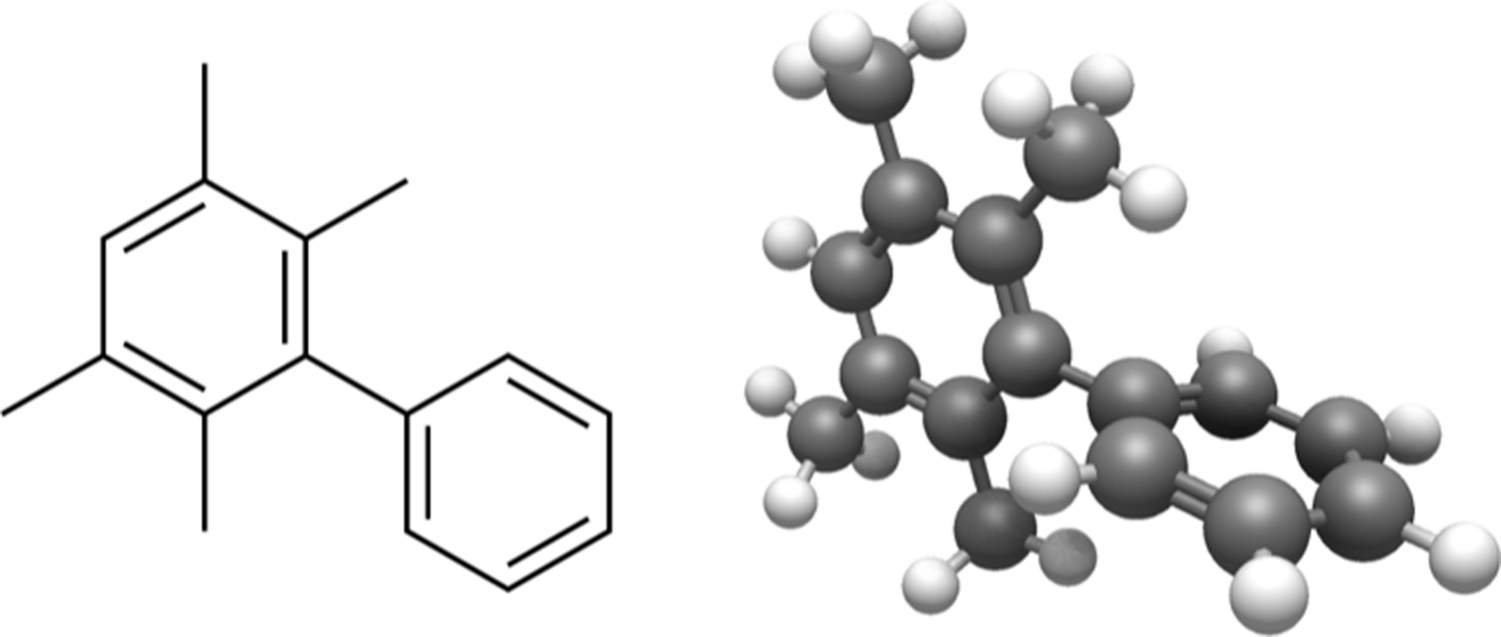
Example of compound matching torsion pattern 270 with steric constraint forcing an angle of ∼90°. Figure from Avogadro2.^67,68^

To test the latter hypothesis, ωB97X-D/def2-SVP geometry optimizations were performed to verify the GFN2-optimized geometries. Five molecules containing a torsion pattern of 270° and an angle of ∼90° were randomly selected from the combined set. The DFT-optimized geometries were found to be in strong agreement (e.g., within a mean absolute deviation of 1.54°) with the GFN2 torsion angles. Such compounds include steric constraints restricting the torsion to ∼90°, as illustrated by Figure 8.

We compared ten random patterns with obvious subpopulations (e.g., 90° in Figure 7), including patterns 10, 23, 48, 53, 125, 149, 216, 253, 270, and 306. For each pattern, five molecules were selected, with the exception of pattern 23, which had only one molecule in the subpopulation. As illustrated in Figure S10, overall absolute torsion angle deviations between the GFN2-optimized and ωB97X-D/def2-SVP-optimized geometries were between 0 and 5°, with the median absolute deviation of 2.89° and a mean absolute deviation of 4.88°.

Comparing across torsion patterns, Table S3 and Figure S11 indicate that some patterns, particularly 48 and 253, reflect larger absolute torsion angle differences of 15 and 10°, respectively, but the overall *r*^2^ correlation between GFN2-optimized and ωB97X-D/def2-SVP torsion angles is high, even for these patterns.

Consequently, while GFN2 is an approximate semiempirical method and correlations with more accurate quantum chemical methods are imperfect,^21^ we can conclude that differences between the crystallographic and gas-phase torsional preferences appear to be mainly derived from lack of data and the presence of new subpopulations in the combined set.

### Fitting and Sampling Torsion Angles

The ETKDG methods derive torsional preferences using a potential energy term fit to a discrete cosine Fourier analysis.^6,7^
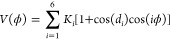
1in which *K* is a force constant and *d* is a phase shift and limited to 0 and π arbitrarily. Consequently, we compare the original published ETDKG fits to a similar cosine analysis of our data. In our work, the phase shift is also varied as a parameter by using nonlinear curve fitting. Note that for both the ETKDG and cosine fits, the histogram probabilities must be transformed into relative energies before fitting. We compare the ETKDG and cosine fits to a sum of up to six Gaussian peaks. The code for the nonlinear curve fitting, via SciPy,^69^ is included at https://github.com/hutchisonlab/quantum-torsions.

**Figure 9:**
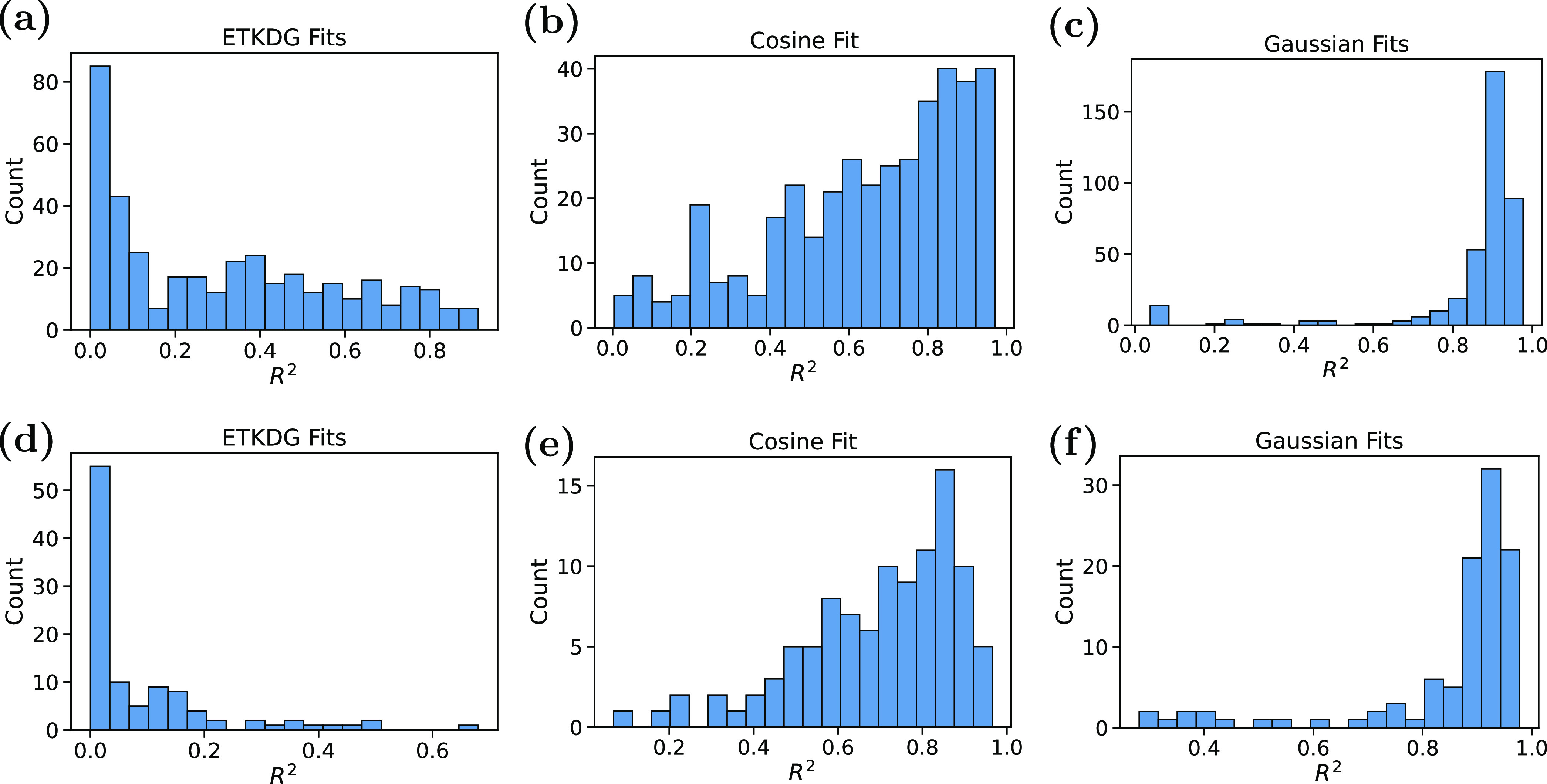
Histograms of correlation between (a,d) ETKDG, (b,e) cosine fits, or (c,f) Gaussian fits and derived torsional histograms for (a–c) acyclic and (d–f) ring torsional patterns.

Compiled in Figure 9 are histograms of correlations among the ETKDG, cosine, and Gaussian fits and the underlying torsional distributions for both acyclic and ring patterns. The Pearson *r*^2^ correlation is used since the magnitudes of the peak heights may vary, but the key features should be the relative intensities at each torsion angle.

Table 1 indicates the median *r*^2^ for each fit across both acyclic and ring patterns, comparing the correlation of relative probabilities at each torsion angle between the fit and the underlying histogram. Note that since the cosine fits in this work allow the phase shift parameter to vary, the results are improved over the published ETKDG fits. We speculate that the fixed phases in the previous ETKDG fits are insufficiently physical, and while it may be useful conceptually to consider these distributions as arising from a typical cosine series potential energy function, this assumption does not fit the present data well.

**Table 1:** Median *r*^2^ Correlation between Fit Functions and the Underlying Quantum Torsions for Both Acyclic and Ring Patterns

method	median acyclic *r*^2^	median ring *r*^2^
ETKDG	0.26	0.03
cosine fits	0.71	0.73
Gaussian fits	0.91	0.93

Nevertheless, simple fits to Gaussian peaks perform noticeably better, with most fits yielding *r*^2^ values above 0.9. In part, this method enables better fits of subpopulations, as discussed above. Further refinement of torsion patterns may resolve such subpopulations, improving the accuracy of ETKDG-style fits.^5^

For sampling torsion angles from the Gaussian fits, we have generated the cumulative sum across all angles from 0 to 360°, normalized and inverted such that generating a uniform random number yields an appropriate torsion angle for a given torsion pattern. Such distributions can either be used for individual torsion driving, as demonstrated in the included script, or for Bayesian sampling,^70,71^ or as part of distance geometry coordinate generation methods such as ETKDG.

Finally, we note that while most previous work has compared computed conformers with experimental crystal structures or bound-ligand geometries, recent work has used cross-sectional areas^14,15^ and rotational spectroscopy^16−18^ to gain geometric insight into gas-phase conformer geometries. Future work should consider such alternate benchmarks since bound conformations, in particular, reflect stabilization from intermolecular interactions.^59^

## Conclusions

Methods such as CREST/GFN2 require a median of 1 h per compound, even using a relatively fast semiempirical quantum method. Consequently, its use for widespread conformational sampling will be limited. Deriving data from experimental crystallographic databases, however, requires far more than 1 h per novel compound to synthesize, crystallize, and analyze via X-ray diffraction and is biased by the need for synthesis and crystallization. This work analyzes over 3 M unique compounds, over twice the reported size of the Cambridge Crystallographic Database.

Overall, comparing geometric RMSD between experimental small-molecule crystal structure geometries and gas-phase GFN2 conformer ensembles indicates a high fidelity compared to the widely used ETKDG methods. Particularly for molecules with few rotatable bonds, the CREST/GFN2 ensembles yielded smaller RMSD than ETKDG and comparable performance across larger molecules from the Crystallographic Open Database. We note somewhat worse performance across the Platinum set than that of ETKDG, particularly for molecules with more rotatable bonds. There is a notable difference in the radius of gyration between the Platinum and low-energy CREST/GFN2 compounds, particularly with increasing numbers of rotatable bonds, likely indicating some preference for extended conformations in the Platinum set due to intermolecular interactions of bound ligands, not present in an isolated gas-phase calculation.^59^

Comparing individual torsion preferences between the experimental COD geometries and the lowest-energy CREST/GFN2 conformers indicated good correlation (i.e., median *r*^2^ above 0.6 for acyclic torsions and above 0.8 for ring torsions), with most cases of poor correlation derived from few examples in the COD set.

The advantage of gas-phase conformer sampling, particularly with the semiempirical GFN2 method, is that many compounds can be analyzed beyond those available in crystallographic databases. Consequently, distributions of torsion preferences were analyzed across over 3 million compounds compiled from multiple small-molecule sets.

Although some differences in torsional preferences were found, most cases of poor correlation between experimental COD geometries and lowest-energy CREST/GFN2 conformers occur with a lack of sufficient experimental crystallographic data. Some deviations occur from new subpopulations not present in the experimental databases as not every compound can be crystallized.^13^

Using density functional methods to optimize compounds in such subpopulations, we find torsion angle deviations of ∼0–5°, suggesting that CREST/GFN2 is sufficiently accurate for generating overall torsion angle distributions, even if some errors remain.

Finally, fits to Gaussian peaks were used to generate sampling distributions and proved to be more accurate than the Fourier analysis used in ETKDG, likely due to the presence of such subpopulations. Further refinement of torsion patterns will be useful,^5^ as well as analysis of correlated torsional preferences for both acyclic and ring torsions.^44,71^

This work has demonstrated the ability of CREST/GFN2 to provide accurate and reliable torsional preferences that could provide a basis for the quantum torsion distance geometry (QTDG) method. Such a method could provide an alternative to ETKDG that does not rely on experimental crystal structure elucidation as well as a method designed particularly for conformer sampling for gas-phase applications rather than targeting crystal structure or bound-ligand geometries that include intermolecular interactions. Finally, QTDG could be used as part of the automated refinement of torsion patterns^5^ since additional conformer sampling can be performed to refine patterns with sparse representation in experimental databases, including charged, novel, or hard-to-crystallize species.^13^ Future work can also carry out comparisons with geometric information from gas-phase spectroscopy.^14−18^

## Supporting Information Available

Histograms of the rotatable bonds and conformers in the COD set, comparisons of RMSD and radius of gyration for the COD and Platinum sets, and histograms of torsional angle deviations for GFN2-optimized and ωB97X-D3/def2-SVP-optimized geometries (PDF)

## Notes

Raw data, Python notebooks, and torsion pattern figures can be found at https://github.com/hutchisonlab/quantum-torsions. All optimized geometries from CREST/GFN2 are available on Figshare https://doi.org/10.6084/m9.figshare.21395061.v1.
