# Supplementary material for: Systematic Comparison of Experimental Crystallographic Geometries and Gas-Phase Computed Conformers for Torsion Preferences
Source: J Chem Inf Model. 2023 Nov 24;63(23):7401–11. doi: 10.1021/acs.jcim.3c01278 (PMC10716907; doi:10.1021/acs.jcim.3c01278)
Supplement: Supplementary file 1 — ci3c01278_si_001.pdf [file ci3c01278_si_001.pdf]

Supporting Information: Systematic Comparison of Experimental Crystallographic Geometries and Gas-Phase Computed Conformers for Torsion Preferences

Dakota L. Folmsbee,[†,‡] David R. Koes,[¶] and Geoffrey R. Hutchison[*,†,§]

†*Department of Chemistry, University of Pittsburgh, 219 Parkman Avenue, Pittsburgh, PA 15260*

‡*Department of Anesthesiology & Perioperative Medicine, School of Medicine, University of Pittsburgh, Pittsburgh, Pennsylvania 15261, United States*

¶*Department of Computational & Systems Biology, School of Medicine, University of Pittsburgh, Pittsburgh, Pennsylvania 15260, United States*

§*Department of Chemical & Petroleum Engineering, University of Pittsburgh, 3700 O'Hara Street, Pittsburgh, PA 15261*

E-mail: geoffh@pitt.edu

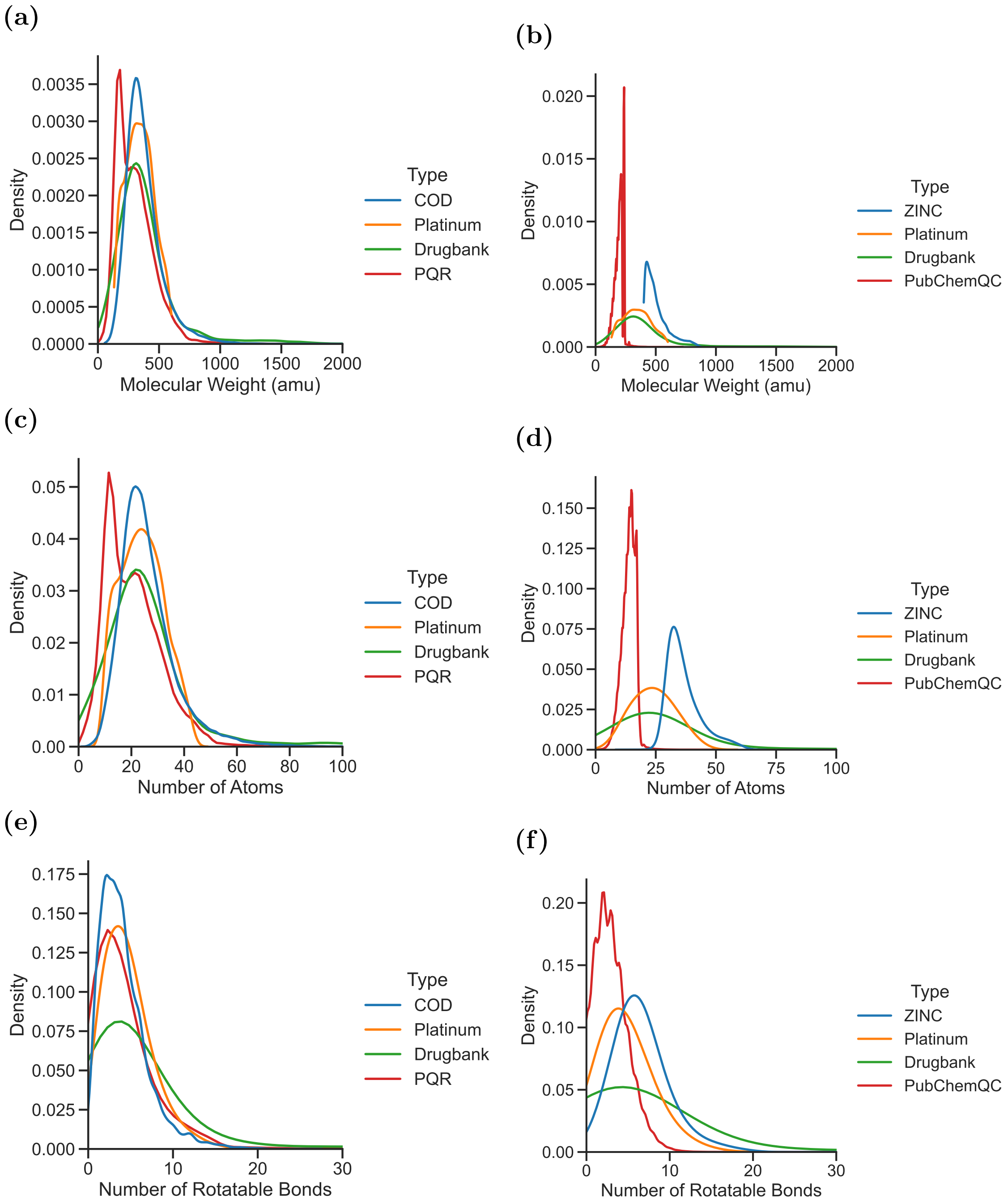

Figure S1: Kernel density histograms for Crystallographic Open Database (COD), Platinum Diverse set, Drugbank Approved, Pitt Quantum Repository (PQR), ZINC subset, and PubChemQC molecular sets including (a, b) molecular weight, (c,d) number of atoms and (e,f) number of rotatable bonds.

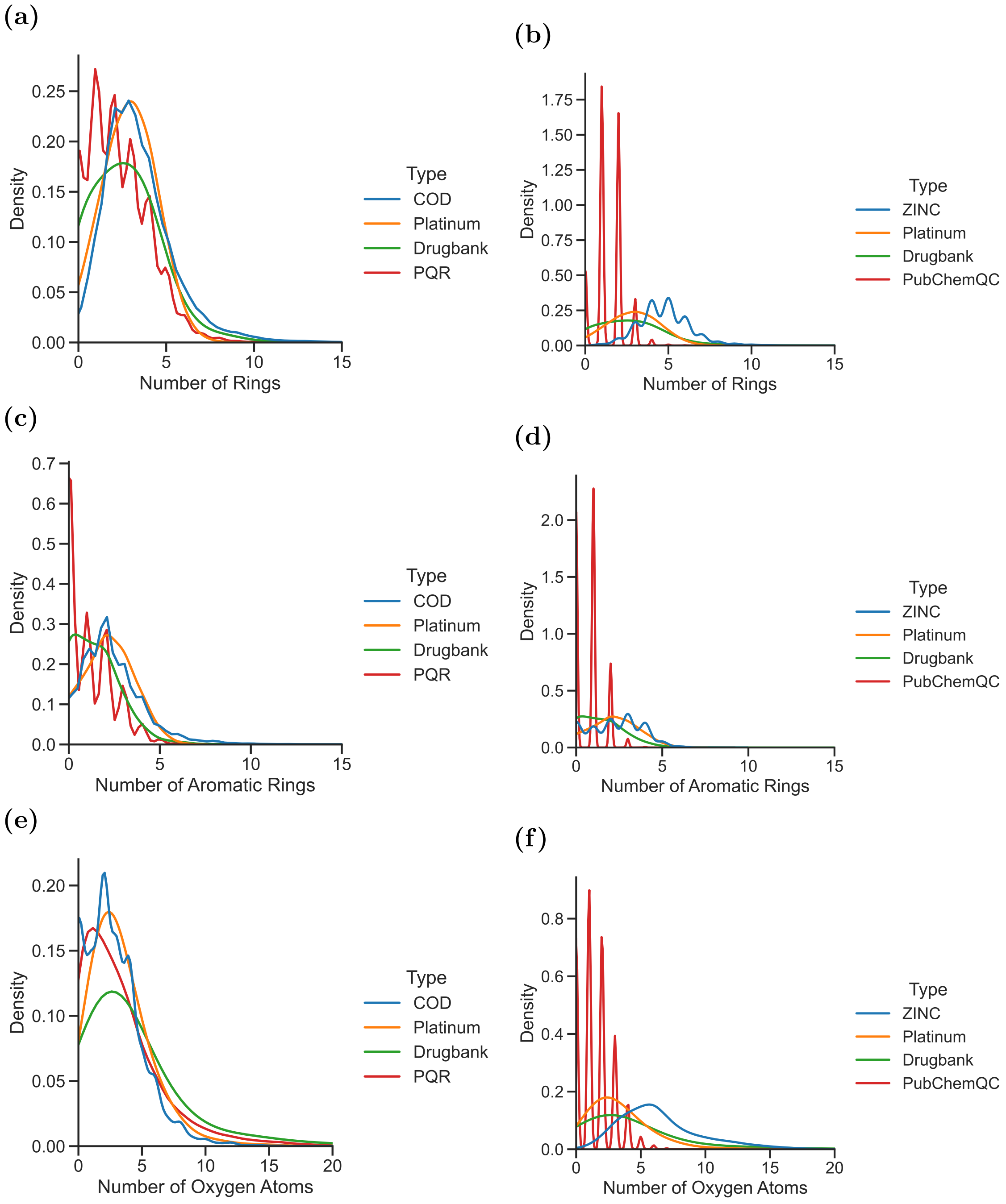

Figure S2: Kernel density histograms for Crystallographic Open Database (COD), Platinum Diverse set, Drugbank Approved, Pitt Quantum Repository (PQR), ZINC subset, and PubChemQC molecular sets including (a, b) number of rings, (c,d) number of aromatic rings and (e,f) number of oxygen atoms.

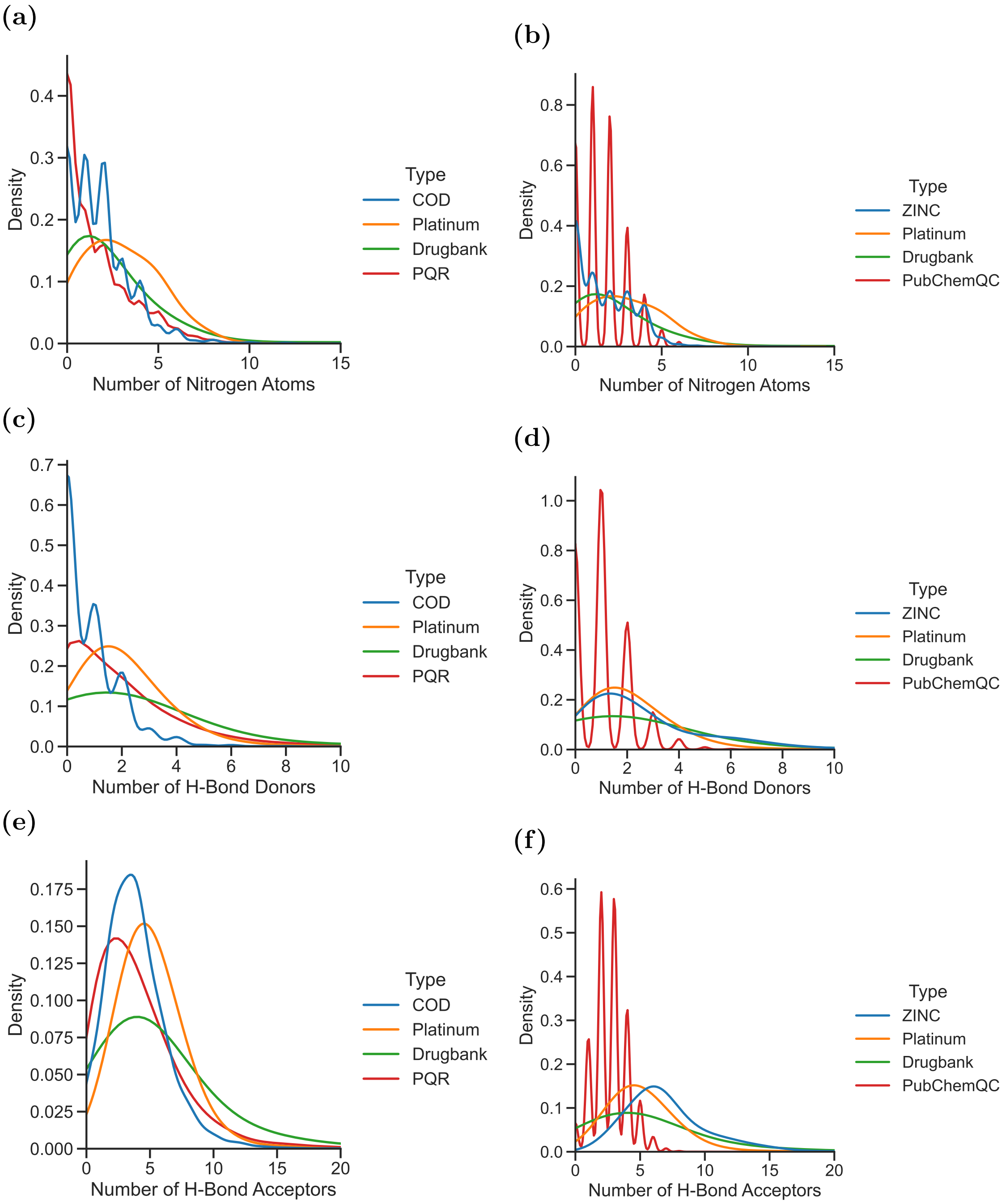

Figure S3: Kernel density histograms for Crystallographic Open Database (COD), Platinum Diverse set, Drugbank Approved, Pitt Quantum Repository (PQR), ZINC subset, and PubChemQC molecular sets including (a, b) number of nitrogen atoms, (c,d) number of hydrogen bond donors and (e,f) number of hydrogen bond acceptors.

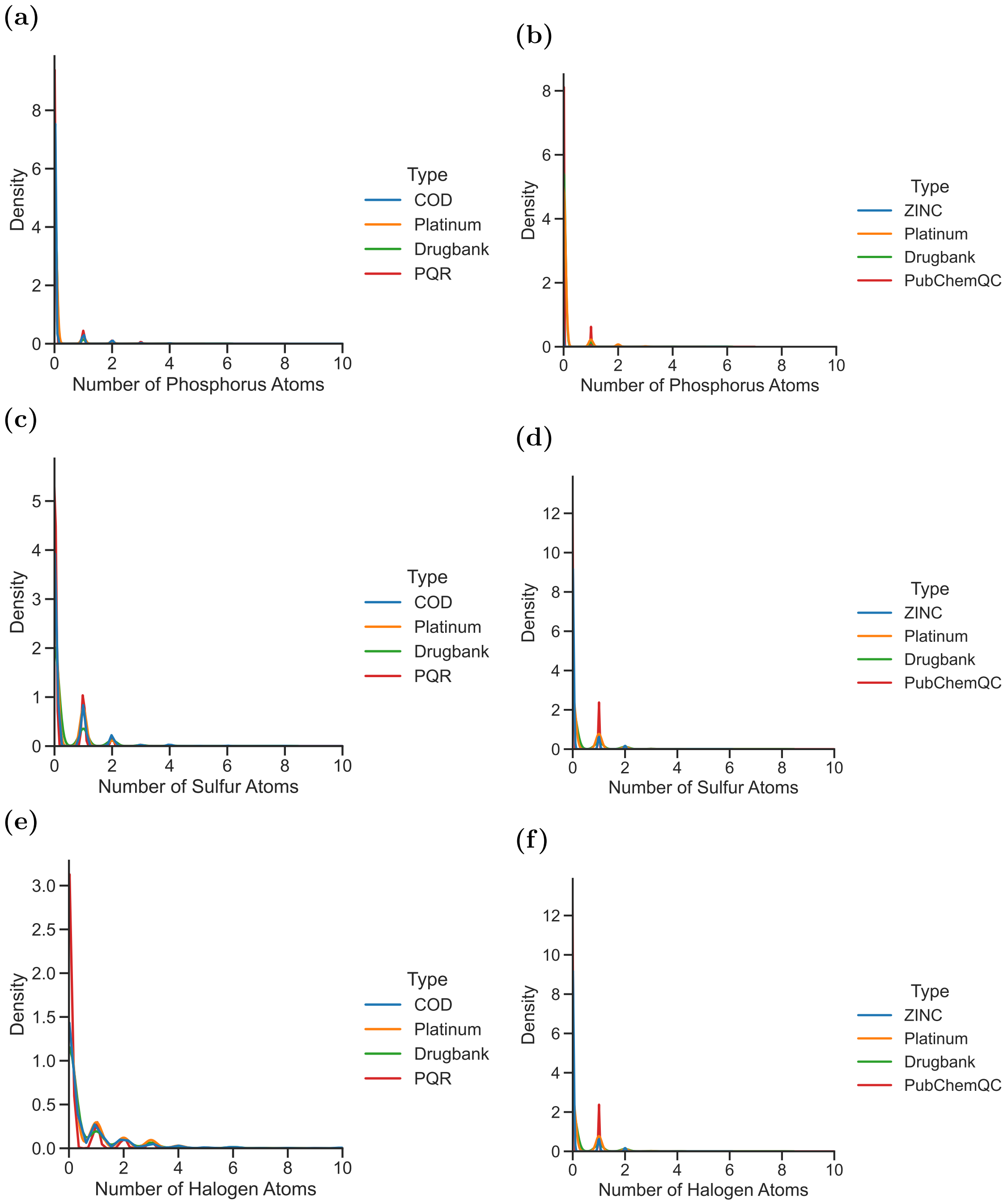

Figure S4: Kernel density histograms for Crystallographic Open Database (COD), Platinum Diverse set, Drugbank Approved, Pitt Quantum Repository (PQR), ZINC subset, and PubChemQC molecular sets including (a, b) number of phosphorus, (c,d) number of sulfur and (e,f) number of halogen atoms.

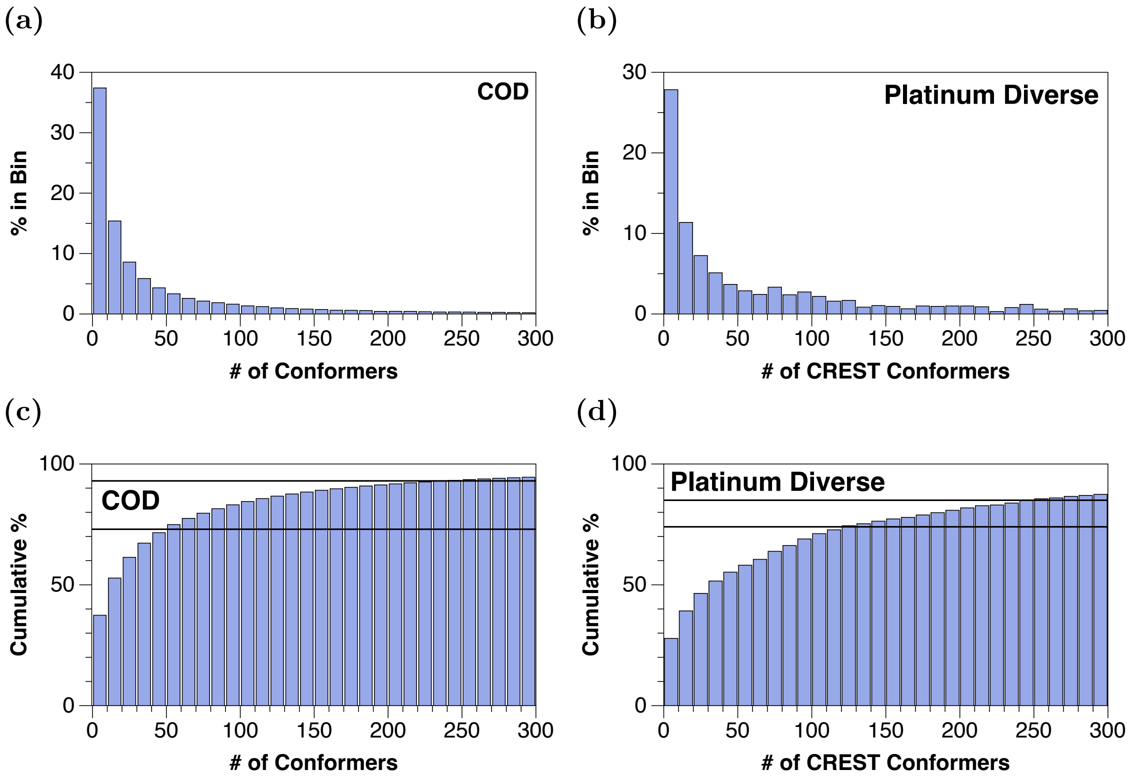

Figure S5: Histograms of # of conformers generated via CREST for (a) the 88,106 organic compounds in the Crystallographic Open Database (COD) and (b) the Platinum Diverse set, and cumulative probabilities indicating the fraction of compounds with under 50 and 250 conformers for (c) the COD and (d) the Platinum diverse set). Note that 93% of the COD and 85% of the Platinum Diverse set have fewer than 250 CREST conformers within 6 kcal/mol of the global minimum, and 73% and 74% of the COD and Platinum Diverse set have fewer than 50 low-energy conformers.

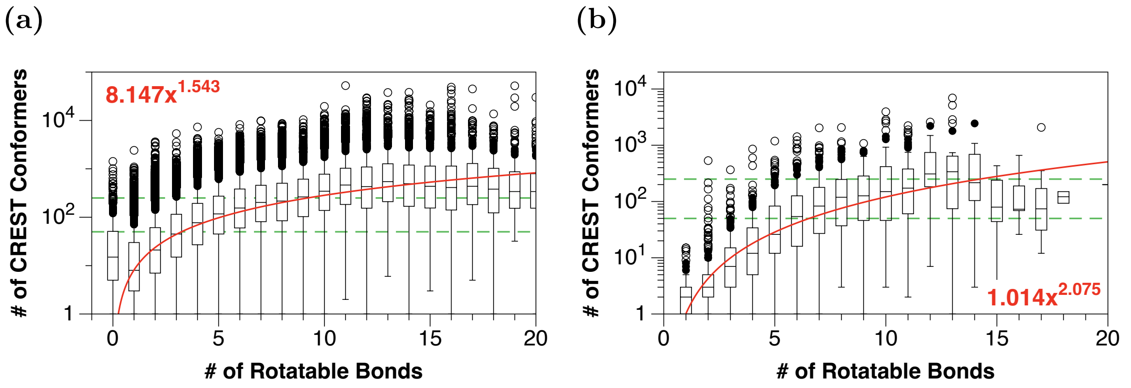

Figure S6: Boxplots of # of conformers generated via CREST for (a) the 88,106 organic compounds in the Crystallographic Open Database (COD) and (b) the Platinum Diverse set as a function of the number of rotatable bonds, indicating sub-exponential power-law fits for the median # of conformers. Dashed green horizontal lines indicate 50 and 250 conformers, respectively.

Table S1: Fraction of molecules successfully reproduced within a specified RMSD threshold. CREST ensembles use default settings including GFN2 minimization. ETKDG ensembles use 250 conformers, followed by UFF minimization.

**Accuracy (Å)**	**0.2**	**0.5**	**1.0**	**1.5**	**2.0**
**COD**					
CREST	0.24	0.68	0.91	0.96	0.98
ETKDGv3	0.22	0.66	0.93	0.98	0.99
**Platinum Diverse**					
CREST	0.12	0.45	0.74	0.86	0.92
ETKDGv3	0.14	0.49	0.89	0.97	0.99

Table S2: Mean and median RMSD (in Å) for Platinum and COD datasets using CREST ensembles with default settings including GFN2 minimization, and ETKDG 250 conformer ensemble followed by UFF minimization.

	**Median RMSD**	**Mean RMSD**
**COD**		
CREST	0.351	0.481
ETKDGv3	0.367	0.453
**Platinum Diverse**		
CREST	0.559	0.815
ETKDGv3	0.508	0.587

Table S3: Summary of torsion angle deviations between GFN2 optimized and ωB97X-D3/def2-SVP optimized geometries for ten acyclic torsion patterns indicated, with mean signed delta (in °), mean absolute deviation (in °) and r^2 correlation.

**Pattern**	**Matches**	**Mean Delta**	**MAD**	r^2
**10**	12	-0.56	2.38	1.00
**23**	1	5.27	5.27	–
**48**	5	-9.53	15.02	0.85
**53**	5	-0.04	2.68	1.00
**125**	5	1.63	6.44	1.00
**149**	8	2.32	6.34	0.99
**216**	15	0.75	3.49	1.00
**253**	5	-10.21	10.54	0.99
**270**	5	0.16	1.54	1.00
**306**	16	4.51	6.05	0.99

(a) COD

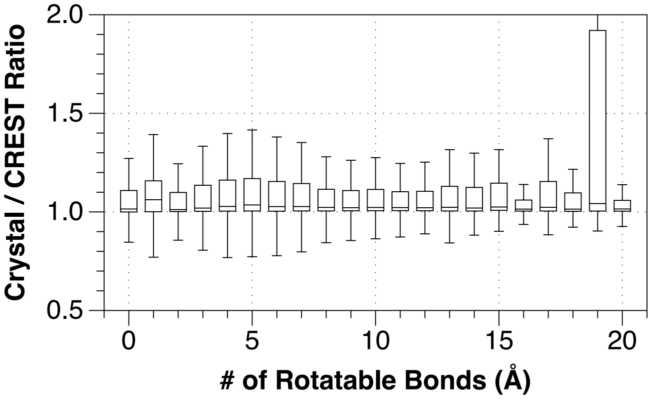

(b) Platinum Set

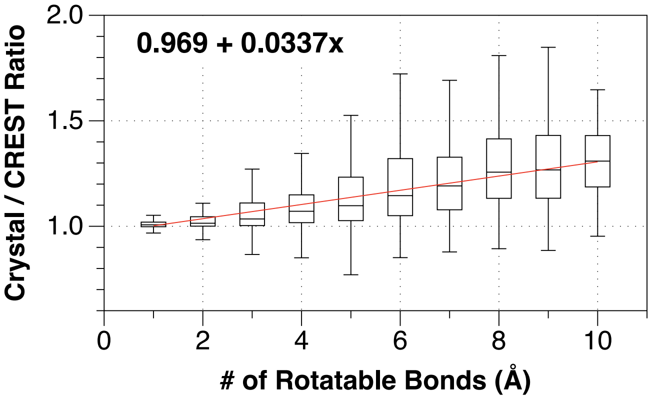

Figure S7: Boxplots of the ratio of computed radius of gyration between (a) COD crystal geometry or (b) Platinum crystal geometry and optimized CREST/GFN2 geometry as a function of the number of rotatable bonds.

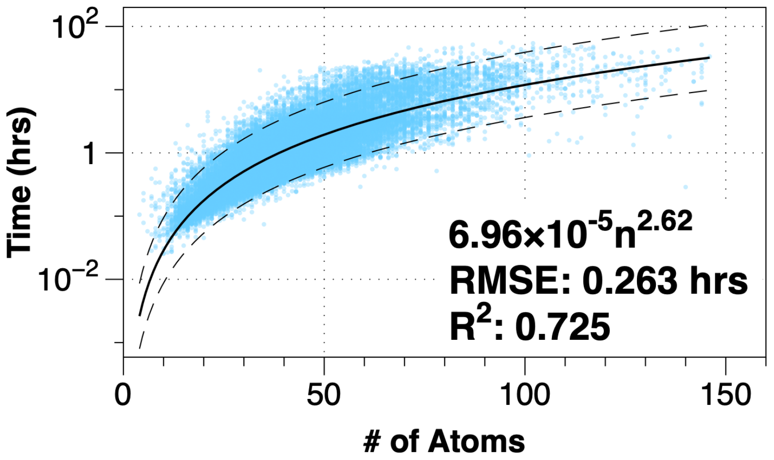

Figure S8: Single-core computational time required for CREST conformer ensemble runs as a function of the number of atoms on the Crystallographic Open Database. Median runtime is 1.0 hours, with approximate fit and root mean square error of the fit and r^2 indicated (e.g., median runtime from ca. 45-75 minutes.

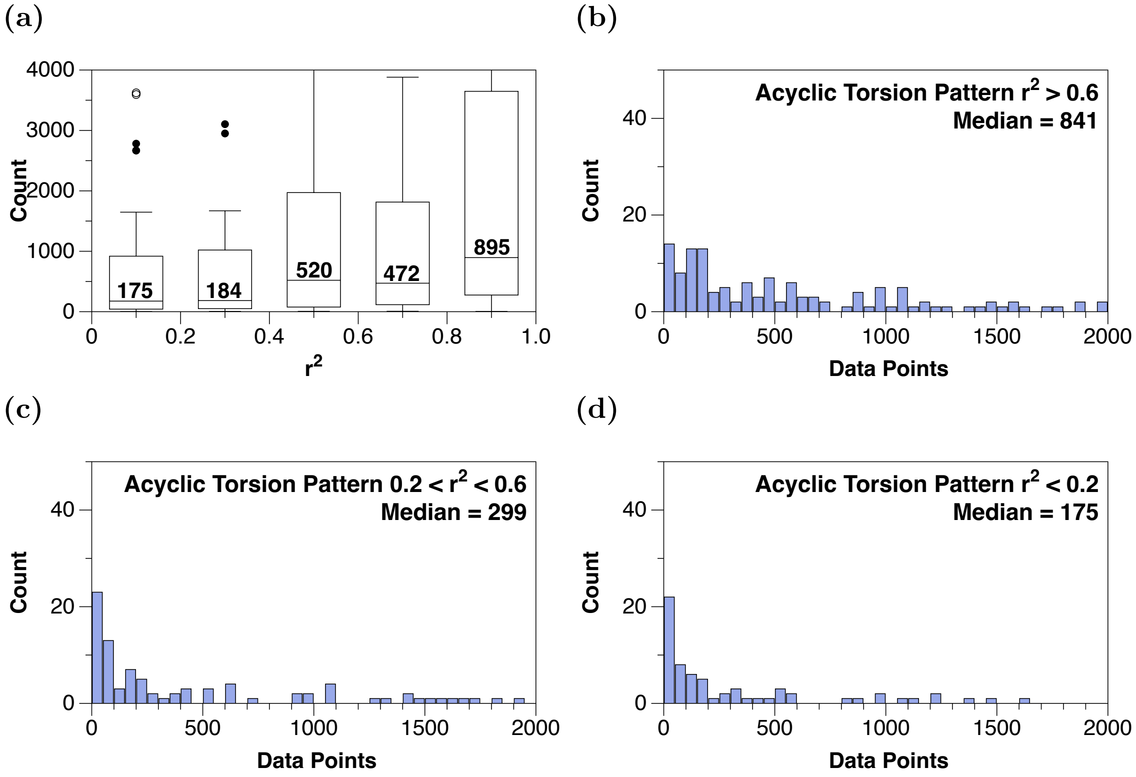

Figure S9: (a) Boxplot of r^2 correlation between experimental and computed gas-phase CREST/GFN2 torsion preferences showing the correlation coefficient r^2 as a function of the number of matching molecules for that torsion pattern. (b-d) Histograms of number of matching data points for a torsion pattern, based on high correlation r^2 between experimental and computed gas-phase torsion preferences, moderate correlation, and low correlation. Note that the median of ca. 175-184 data points is found for patterns exhibiting poor correlation.

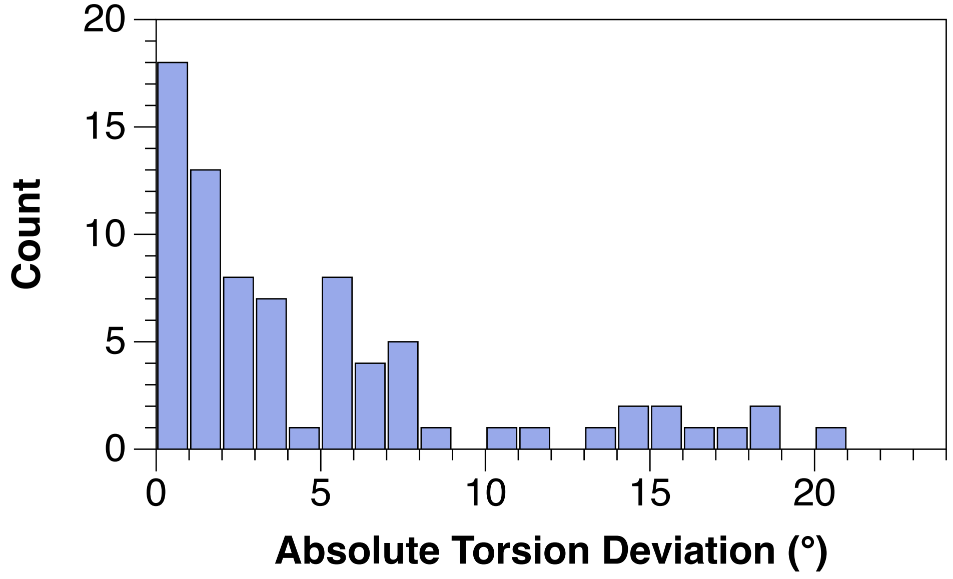

Figure S10: Histogram of absolute torsion angle deviations between GFN2-optimized and ωB97X-D/def2-SVP optimized geometries.

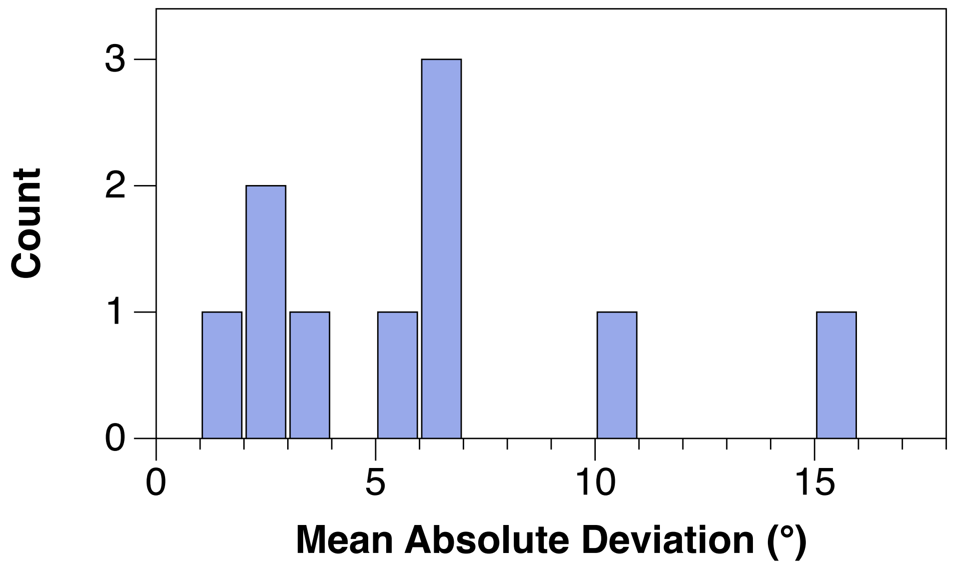

Figure S11: Histogram of mean absolute torsion angle deviations between GFN2-optimized and ωB97X-D/def2-SVP optimized geometries, across ten acyclic torsion patterns.
